# Hepatic venous pressure gradient in patients with (compensated and decompensated) advanced chronic liver disease – A comparison of metabolic dysfunction-associated steatotic liver disease with alcohol-associated liver disease: A retrospective view

**DOI:** 10.1371/journal.pone.0317287

**Published:** 2025-03-05

**Authors:** Ĺubomír Skladaný, Daniela Žilinčanová, Michal Žilinčan, Stanislav Okapec, Filip Danček, Svetlana Adamcová-Selčanová, Michal Kukla, Tomáš Koller

**Affiliations:** 1 Department of Hepatology, Gastroenterology, and Transplantation, 2nd Department of Medicine, Slovak Medical University Faculty of Medicine, F. D. Roosevelt Hospital, Banska Bystrica, Slovakia; 2 Department of Radiology, FD Roosevelt Hospital, Banská Bystrica, Slovakia; 3 Department of Internal Medicine and Geriatrics, Faculty of Medicine, Jagiellonian University Medical College, Kraków, Poland; 4 Department of Endoscopy, University Hospital in Kraków, Kraków, Poland; 5 Subdivision of Gastroenterology and Hepatology, 5th Department of Medicine, Comenius University Faculty of Medicine, University Hospital Bratislava, Bratislava, Slovakia; Institute for Clinical and Experimental Medicine, CZECHIA

## Abstract

**Background and aims:**

Hepatic venous pressure gradient (HVPG) is a strong surrogate of severity and outcome but its relative prognostic value in metabolic dysfunction-associated steatotic liver disease (MASLD) and alcohol-associated liver disease (ALD) is yet to be clarified. We compared HVPG in MASLD with ALD and other etiologies according to cirrhosis complications.

**Patients and methods:**

In our cirrhosis registry RH7, we identified patients with data on HVPG and scrutinized them against the etiology of advanced chronic liver disease (ACLD) (MASLD, ALD, Other) and specific complications of ACLD such as variceal bleeding or ascites. We excluded patients with advanced malignancies and less than 6 months of follow-up.

**Results:**

We enrolled 220 patients with ALD, MASLD, and Other etiology in 128, 52, and 40 cases, respectively; te median age was 57, 60, and 52 years (P = 0.09); the proportion of females was 31, 67, and 55%, respectively (P < 0.01). Median MELD scores in ALD, MASLD, and Other etiologies were 16.0, 13.0, and 12.0 (P < 0.01), and the median HVPG was 18.0, 14.0, and 11.5 mmHg (P < 0.001). In 19, 30, and 25 compensated patients, the median HVPG was 10.0, 11.5, and 11.0 mmHg (P = 0.97). In 109, 22, and 15 decompensated patients, the median HVPG was 19.0, 15.5 and 14 mmHg (P = 0.01 for trend, difference ALD vs. other P < 0.01, ALD vs. MASLD, P = 0.295). Between decompensated MASLD and ALD patients, we observed no differences in the proportion of clinically significant portal hypertension (CSPH) (>10 mmHg).

**Conclusion:**

In our cirrhosis registry study of hospitalized patients with ACLD, baseline HVPG measured for accepted indications differed according to the etiology of dACLD: patients with ALD had the highest values followed by MASLD and Other etiologies. Importantly, when looked at from the point of view of complications, the treshold for clinically significant portal hypertension remained fixed at the level recommended by BAVENO Consensus - 10 mm Hg irrespective of etiology.

## Introduction

In our Central European region, liver diseases are the number one cause of death in young adults from 25-44 years, with alcohol-associated liver disease (ALD) being the most prevalent etiology. With the growing prevalence of obesity, metabolic dysfunction-associated steatotic liver disease (MASLD) is the fastest-growing cirrhosis etiology [1]. In addition, our region ranks number one in the world in the prevalence of compensated and decompensated cirrhosis [2]. To stand up against the challenges posed by this trend, in 2014 we launched the cirrhosis registry (RH7) which has been enrolling consecutive hospitalized patients admitted in the tertiary liver & transplant unit to create the dataset for strategic analyses. We have previously demonstrated that disease etiology impacts the baseline characteristics and prognosis of the registry patients [3] - namely that patients with MASLD (previously known as non-alcoholic fatty liver disease [NAFLD]) had less severe baseline markers of liver decompensation and lower markers of systemic inflammation as compared to ALD; however, paradoxically, their long-term prognosis was worse. Currently, there is an ongoing discussion on how the pathogenesis and natural history of portal hypertension in MASLD differ from other etiologies [4–6]. Authors have reported lower HVPG values in advanced MASLD as compared with chronic hepatitis C, or ALD. There is also an ongoing discussion on the reasons for the observed differences, some authors attribute them to underestimation of the true portal pressure by the hepatic venous pressure gradient (HVPG) due to the different patterns of perisinusoidal fibrosis. Yet, the precise mechanisms have not been sufficiently clarified [7] and our understanding of the unique pathophysiology of portal hypertension in MASLD remains to be elucidated.

Therefore, we aimed to explore our registry data for the differences in HVPG between MASLD, ALD, and other etiologies – autoimmune or viral hepatitis, as measured in the context of a diagnostic workup in compensated advanced chronic liver disease (cACLD), or specific complications of decompensated advanced chronic liver disease (dACLD): variceal bleeding or ascites.

## Patients and methods

The RH7 cirrhosis registry operates at the academic tertiary liver & transplant unit since 2014 (NCT04767945) [8]. In RH7, we collect baseline data on consecutive adult patients admitted to the hospital with ACLD and its complications: demographics, anthropometry, etiology of ACLD, nutritional status, frailty by the Liver frailty index (LFI), basic liver laboratory parameters and prognostic indices such as the model for the end-stage liver disease (MELD), and, in selected cases, the HVPG. Indications for HVPG measurement were as follows: management of variceal bleeding (hemodynamic response-driven secondary prophylaxis, preemptive or rescue TIPS placement), ascites and/or hepatic hydrothorax (therapeutic TIPS placement), staging of ACLD, decision-making about surgery in hepatocellular carcinoma (HCC), diagnostic work-up with transjugular liver biopsy, etc. Of patients identified in RH7 as having HVPG measurement, we included patients with at least six months of follow-up. *We excluded* patients with incomplete data required for planned analysis, and those with malignancies apart from HCC within the Milan criteria. In patients having HVPG measurement for diagnostic purposes or before liver resection, the disease was considered compensated (cACLD). Disease decompensation (dACLD) was defined by the presence of ascites and/or hepatic hydrothorax, variceal bleeding, hepatic encephalopathy, and severe alcohol-associated hepatitis. Clinically significant portal hypertension (CSPH) was defined according to the BAVENO consensus by HVPG values ≥  10 mmHg [9]. Hepatic encephalopathy (HE) was diagnosed by an experienced clinical hepatologists based on clinical evaluation, number connection test, and the Stroop Test, with supporting evidence from ammonia levels, and was graded according to the West Haven classification [10]. Acute kidney injury (AKI) was defined according to the recent consensus [11]. Patients with HCC were included only when the disease was considered indicated for a curable therapy such as liver resection or liver transplantation (LT) within the Milan criteria. The follow-up was censored at least 6 months from admission to tertiary care, or when patients underwent liver transplantation or have died. Deaths were verified from the National registry of the deceased inhabitants.

All procedures involving human participants have been approved according to the ethical standards of the institutional research committee, including the 1964 Helsinki Declaration and its later amendments (www.wma.net) or comparable ethical standards. The reported clinical and research activities are consistent with the Principles of the Declaration of Istanbul, as outlined in the Declaration of Istanbul on Organ Trafficking and Transplant Tourism. All patients signed informed consent before the HVPG or TIPS procedure. The enrolment in RH7 and data acquisition was approved by the local ethics committee on May 21^st^, 2014.

### HVPG measurement

HVPG measurements were performed in fasting patients in the supine position only with local anesthesia (MZ, SO, FD). The right internal jugular vein was punctured under ultrasound guidance. A special cannula for HVPG measurements and transjugular biopsy was inserted into the right jugular vein. Systemic venous pressure [measured in the infrarenal inferior vena cava (IVC)] was measured with the same catheter. Wedged hepatic venous pressure (WHVP) was measured in the right hepatic vein with the same catheter using the wedge technique. The catheter was placed in the distal part of the hepatic vein, where the size of the catheter exceeded the size of the vessel lumen. Adequate occlusion of the hepatic vein was confirmed by dye injection. Systemic venous pressure and wedge hepatic venous pressure were allowed to stabilize for at least 60 seconds before recording.

### TIPS placement

Procedures were performed under conscious sedation. A right internal jugular vein was the preferred approach. The right jugular vein was accessed under ultrasound guidance. Ten French introducer sheaths were used in all cases. A catheter was then advanced beyond the right atrium into the right hepatic vein under fluoroscopic guidance. A wedged hepatic venogram is performed to visualize the portal vein to allow good access. Colapinto puncture needle; Rosch-Uchida transjugular liver access set (Cook Medical) which contains a 14-gauge needle was used. After visualizing the portogram, the needle was directed from the right hepatic vein to the right branch of the portal vein. After the portal vein access was gained, the needle was removed, and a wire and catheter were advanced into the splenic or mesenteric vein. Portal vein angiography and pressure measurements were performed. The tract between HV and PV was dilated with an angioplasty balloon. After precise measurements, the balloon-expandable stent-graft was deployed into the pre-dilated tract. We used 10mm or 8mm balloon-expandable stent grafts. After stent-graft implantation, pressure measurements were repeated. When the pressure remained higher than desired, the stent was re-dilated with a 10 mm angioplasty balloon.

### Statistical analysis

Results of non-normally distributed variables are displayed as medians and 25-75 percentiles and compared using the Mann-Whitney test, proportions are displayed as numbers and percentages and compared with the chi-square test with P values for trend (Tables 1 and 2). Differences in HVPG among more than two etiological groups were assessed by the Kruskal-Wallis test with a Jackeere-Terpstra test for trend (Table 3). In a cross-sectional analysis at baseline, we also aimed to identify the HVPG threshold for predicting hepatic decompensation according to the disease etiology by using the receiver operating curve analysis (ROC) providing the areas under the curve (AUROC) with a 95% confidence interval according to DeLong et al. [12] (Fig 4). Correlations between numerical parameters were carried out using the Spearman´s rank-correlation for non-parametric data. A significant difference was defined by P values less than 0.05. Statistical analysis was conducted using the package MedCalc® Statistical Software version 20.009 (MedCalc Software Ltd, Ostend, Belgium; www.medcalc.org; 2021) and R (R foundation for statistical computing, Vienna, Austria, https://www.r-project.org/).

**Table 1:** Comparison of baseline characteristics of patients by the disease etiologies.

	Liver disease etiology				
	Alcohol associated (ALD)	Metabolic (MASLD)	Other π	P value#	P value*
N	128	52	40	for trend	ALD vs. MASLD
	Median [25-75P], n (%)				
Age, y	56.86 [45.04, 62.17]	60.42 [47.98, 62.94]	52.74 [33.79, 63.02]	0.092	0.165
Sex, n(%)					
Male	88 (68.8)	17 (32.7)	18 (45.0)	<0.001	<0.001
Female	40 (31.2)	35 (67.3)	22 (55.0)		
Body mass index, kg/m2	25.88 [23.12, 29.84]	26.50 [23.91, 30.37]	24.94 [20.88, 27.00]	0.041	0.411
Type 2 diabetes, n(%)	28 (21.9)	14 (26.9)	3 (7.5)	0.060	0.560
Hand grip strength, kg	26.34 [18.52, 34.08]	18.80 [14.80, 34.72]	23.60 [18.29, 30.75]	0.316	0.112
Mid-arm circumference, cm	26.00 [23.25, 29.00]	29.00 [25.00, 31.50]	26.50 [23.25, 28.75]	0.013	0.006
Tricipital skinfold, mm	10.05 [6.25, 17.15]	17.00 [9.00, 23.10]	12.20 [6.35, 18.40]	0.013	0.003
Hepatic encephalopathy, n(%)					
None	99 (82.5)	44 (86.3)	36 (97.3)	0.307	0.584
Stage 1	18 (15.0)	5 (9.8)	1 (2.7)		
Stage 2	2 (1.7)	2 (3.9)	0 (0.0)		
Stage 4	1 (0.8)	0 (0.0)	0 (0.0)		
Child-Pugh-Turcotte score	9.00 [7.00, 10.00]	7.00 [6.00, 8.75]	8.00 [5.00, 9.00]	0.001	0.001
MELD score	16.00 [12.00, 20.00]	13.00 [8.25, 17.50]	12.00 [9.00, 16.00]	0.003	0.008
Albumin, g/l	28.00 [25.00, 34.00]	33.00 [28.00, 37.00]	31.00 [27.00, 37.25]	0.004	0.003
Bilirubin, umol/l	36.60 [22.10, 86.90]	23.50 [13.95, 43.85]	27.10 [14.65, 54.95]	0.007	0.003
CRP, mg/l	10.75 [4.93, 22.60]	8.68 [3.01, 18.59]	7.02 [1.59, 13.59]	0.023	0.195
Leucocytes, x 10 * 9/l	6.15 [4.50, 8.38]	4.90 [3.25, 6.60]	4.90 [3.70, 7.65]	0.035	0.018
Lymphocytes, x10 * 9/l	1.20 [0.90, 1.70]	1.05 [0.58, 1.50]	1.05 [0.58, 1.52]	0.167	0.085
Beta-adrenergic blocker therapy	79 (61.7)	29 (55.8)	21 (52.5)	0.522	0.504
Indication for HVPG, n(%)					
Alcoholic hepatitis	27 (21.1)	NA	NA		
Ascites	31 (24.2)	6 (11.5)	3 (7.5)	0,021	0,057
Bleeding	51 (39.8)	16 (30.8)	12 (30.0)	0,356	0,255
Compensated, diagnosis only	19 (14.8)	30 (57.7)	25 (62.5)	<0.001	<0.001
Endpoints during follow-up, n(%)					
Death or liver transplantation	77 (60.20)	18 (34.60)	24 (60.0)	0.006	0.002
Endpoint in 30 days, n(%)	9 (7.0)	5 (9.6)	4 (10.0)	0.762	0.558
Endpoint in 90 days, n(%)	19 (14.8)	8 (15.4)	9 (22.5)	0.508	0.929
Endpoint in 180 days, n(%)	31 (24.2)	9 (17.3)	12 (30.0)	0.354	0.313
Follow-up, days	448.00 [201.0, 1045.0]	696.0 [386.25, 943.50]	368.0 [118.00, 1029.75]	0.244	0.141

*) p value for comparison of alcoholic vs. metabolic, π) autoimmune, primary biliary or primary sclerosing cholangitis, viral hepatitis

#) p value for difference among the three groups

**Table 2:** Comparison of disease characteristics, HVPG and outcomes by disease phenotype and events.

	n (%) or median [25-75 percentil]			
	Compensated	Decompensated		
		Alcoholic hepatitis	Refractrory ascites	Bleeding
N	74	27	40	79
Age, y	56.88 [43.79, 63.55]	43.10 [37.14, 50.84]	59.42 [53.31, 62.38]	58.73 [45.36, 63.18]
Sex, n (%) men	37 (50.0)	16 (59.3)	26 (65.0)	44 (55.7)
Women	37 (50.0)	11 (40.7)	14 (35.0)	35 (44.3)
MELD score	13.00 [9.00, 17.00]	26.00 [20.00, 29.00]	13.00 [10.00, 17.00]	14.00 [10.25, 18.00]
Child-Pugh-Turcotte score	7.00 [5.00, 9.00]	10.00 [9.00, 12.00]	9.00 [7.75, 10.00]	8.00 [6.00, 10.00]
Beta-adrenergic blocker therapy	40 (54.1)	11 (40.7)	28 (70.0)	50 (63.3)
Hepatic venous pressure gradient (HVPG), mmHg	11.00 [7.00, 16.00]	16.00 [13.00, 19.50]	19.00 [16.00, 22.25]	18.00 [14.00, 22.00]
HVPG ≥ 10 mmHg, n (%)	46 (62.2)	26 (96.3)	39 (97.5)	73 (92.4)
HVPG ≥ 12 mmHg, n (%)	34 (45.9)	22 (81.5)	37 (92.5)	67 (84.8)
HVPG ≥ 16 mmHg, n (%)	23 (31.1)	14 (51.9)	31 (77.5)	52 (65.8)
HVPG ≥ 20 mmHg, n (%)	10 (13.5)	7 (25.9)	18 (45.0)	31 (39.2)
Transjugular porto-systemic shunt placement	1 (1.4)	2 (7.4)	33 (82.5)	35 (44.3)
Death or liver transplantation, n (%) within 30 days	4 (5.4)	2 (7.4)	2 (5.0)	10 (12.7)
within 90 days	9 (12.2)	8 (29.6)	3 (7.5)	16 (20.3)
within 180 days	14 (18.9)	12 (44.4)	5 (12.5)	21 (26.6)
within 365 days	19 (25.7)	12 (44.4)	10 (25.0)	28 (35.4)

**Table 3:** Comparison of HVPG values, tresholds and TIPSS placement by disease etiology and events.

All cases					
	Alcohol associated (ALD)	Metabolic (MASLD)	Other*	P value	P value
	n (%) or median [25, 75 percentil]			for trend	ALD vs. MASLD
N	128	52	40		
HVPG, mmHg	18.00 [13.75, 22.00]	14.00 [9.00, 20.00]	11.50 [9.00, 17.00]	<0.001	0,009
HVPG ≥ 10, n (%)	117 (91.4)	38 (73.1)	29 (72.5)	0.001	0,001
HVPG ≥ 12, n (%)	107 (83.6)	33 (63.5)	20 (50.0)	<0.001	0,005
HVPG ≥ 16, n (%)	83 (64.8)	23 (44.2)	14 (35.0)	0.001	0,013
HVPG ≥ 20, n (%)	48 (37.5)	14 (26.9)	4 (10.0)	0.004	0,226
Beta-adrenergic blocker, n (%)	79 (61.7)	29 (55.8)	21 (52.5)	0.552	0,504
TIPSS placement, n (%)	52 (40.6)	15 (28.8)	4 (10.0)	0.001	0,14
**Compensated disease**					
	Alcohol associated (ALD)	Metabolic (MASLD)	Other*		
N	19	30	25		
HVPG, mmHg	10.00 [7.50, 13.50]	11.50 [6.25, 17.00]	11.00 [9.00, 16.00]	0.975	0,73
HVPG ≥ 10, n (%)	12 (63.2)	17 (56.7)	17 (68.0)	0.685	0,665
HVPG ≥ 12, n (%)	8 (42.1)	15 (50.0)	11 (44.0)	0.840	0,769
HVPG ≥ 16, n (%)	4 (21.1)	12 (40.0)	7 (28.0)	0.347	0,219
HVPG ≥ 20, n (%)	2 (10.5)	6 (20.0)	2 (8.0)	0.392	0,458
Beta-adrenergic blocker, n (%)	10 (52.6)	17 (56.7)	13 (52.0)	0.932	0,784
TIPSS placement, n (%)	0 (0.0)	1 (3.3)	0 (0.0)	0.476	NA
**Decompensated disease**					
	Alcohol associated (ALD)	Metabolic (MASLD)	Other*		
N	109	22	15		
HVPG, mmHg	19.00 [15.00, 22.00]	15.50 [13.25, 22.75]	14.00 [10.00, 17.50]	0.019	0,296
HVPG ≥ 10, n (%)	105 (96.3)	21 (95.5)	12 (80.0)	0.033	0,84
HVPG ≥ 12, n (%)	99 (90.8)	18 (81.8)	9 (60.0)	0.004	0,252
HVPG ≥ 16, n (%)	79 (72.5)	11 (50.0)	7 (46.7)	0.029	0,039
HVPG ≥ 20, n (%)	46 (42.2)	8 (36.4)	2 (13.3)	0.096	0,644
Beta-adrenergic blocker, n (%)	69 (63.3)	12 (54.5)	8 (53.3)	0.607	0,442
TIPSS placement, n (%)	52 (47.7)	14 (63.6)	4 (26.7)	0.087	0,175
**Bleeding**					
	Alcohol associated (ALD)	Metabolic (MASLD)	Other*		
N	51	16	12		
HVPG, mmHg	19.00 [16.00, 22.00]	15.50 [13.75, 22.25]	13.50 [10.00, 17.25]	0.014	0,395
HVPG ≥ 10, n (%)	48 (94.1)	15 (93.8)	10 (83.3)	0.436	0,957
HVPG ≥ 12, n (%)	47 (92.2)	13 (81.2)	7 (58.3)	0.012	0,344
HVPG ≥ 16, n (%)	39 (76.5)	8 (50.0)	5 (41.7)	0.024	0,045
HVPG ≥ 20, n (%)	24 (47.1)	6 (37.5)	1 (8.3)	0.047	0,573
Beta-adrenergic blocker, n (%)	34 (66.7)	8 (50.0)	8 (66.7)	0.466	0,232
TIPSS placement, n (%)	34 (66.7)	9 (56.2)	3 (25.0)	0.253	0,44
**Ascites**					
	Alcohol associated (ALD)	Metabolic (MASLD)	Other*		
N	31	6	3		
HVPG, mmHg	19.00 [16.00, 22.00]	16.50 [12.50, 22.75]	16.00 [12.00, 20.00]	0.578	0,386
HVPG ≥ 10, n (%)	31 (100.0)	6 (100.0)	2 (66.7)	0.075	NA
HVPG ≥ 12, n (%)	30 (96.8)	5 (83.3)	2 (66.7)	0.121	0,301
HVPG ≥ 16, n (%)	26 (83.9)	3 (50.0)	2 (66.7)	0.126	0,1
HVPG ≥ 20, n (%)	15 (48.4)	2 (33.3)	1 (33.3)	0.860	0,666
Beta-adrenergic blocker, n (%)	24 (77.4)	4 (66.7)	0	0.02	0,619
TIPSS placement, n (%)	27 (87.1)	5 (83.3)	1 (33.3)	0.093	0,81

* autoimmune, primary biliary or primary sclerosing

**Fig 1:**
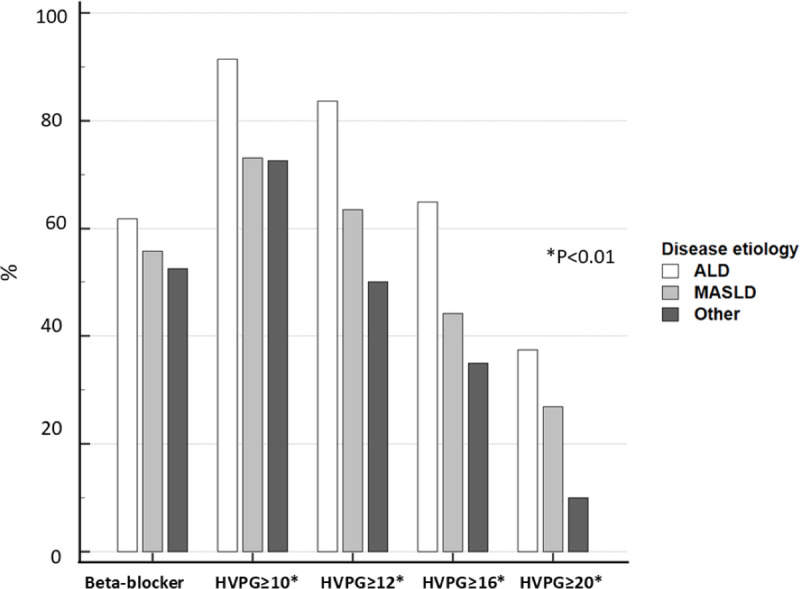
Comparison of proportions of beta-adrenergic blocker therapy and relevant HVPG thresholds among the etiological groups of advanced chronic liver disease in the entire study cohort. N = 220. Alcohol (ALD, white bars), metabolic (MASLD, grey bars), other (black bars). Numerical values are displayed in Table 3. P-value for trend: beta-blocker therapy, P = 0.552, HVPG ≥ 10, 12, 16, 20 mmHg and TIPSS placement, all P < 0.01.

**Fig 2:**
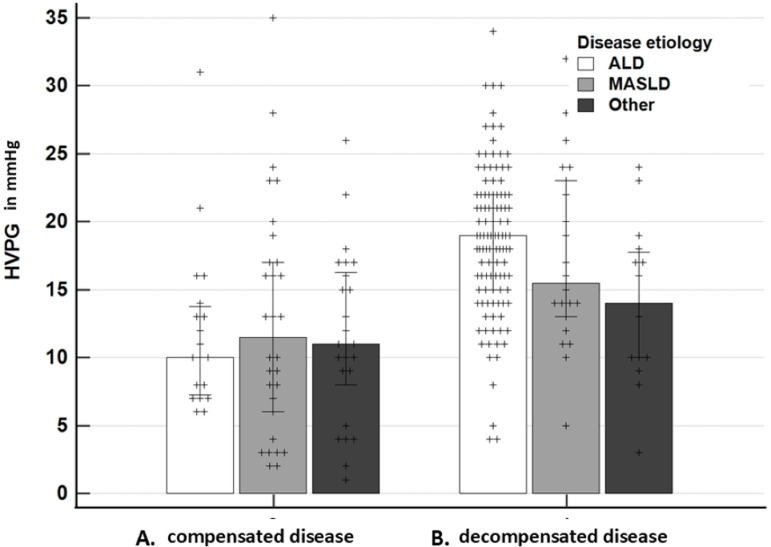
Comparison of the hepatic venous pressure gradients (median HVPG in mmHg, 25-75 percentil) among the etiological groups of advanced chronic liver disease. Alcohol (ALD, white bars), metabolic (MASLD, grey bars), other (black bars), A. Compensated disease, median 10.0 vs. 11.5 vs. 11.0 mmHg, P value for trend = 0.975. B. Decompensated disease, median 19.0 vs. 15.5 vs. 14.0 mmHg, P value for trend < 0.019.

**Fig 3:**
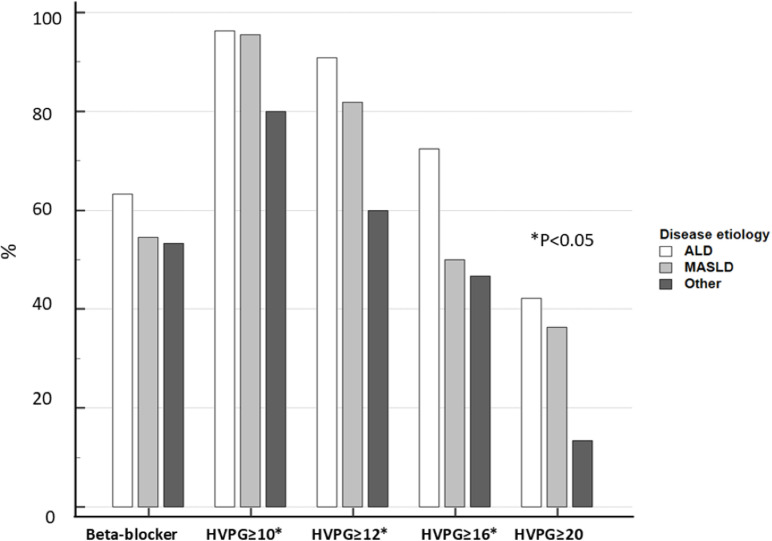
Comparison of proportions of beta-adrenergic blocker therapy and relevant HVPG thresholds among the etiological groups in the subgroup of decompensated patients. N = 146, numerical values are displayed in Table 3. P-value for trend: beta-blocker therapy, P = 0.607, HVPG ≥ 10, 12, 16 mmHg, P < 0.05, ≥ 20 mmHg, P = 0.1, TIPSS placement, P = 0.09.

**Fig 4:**
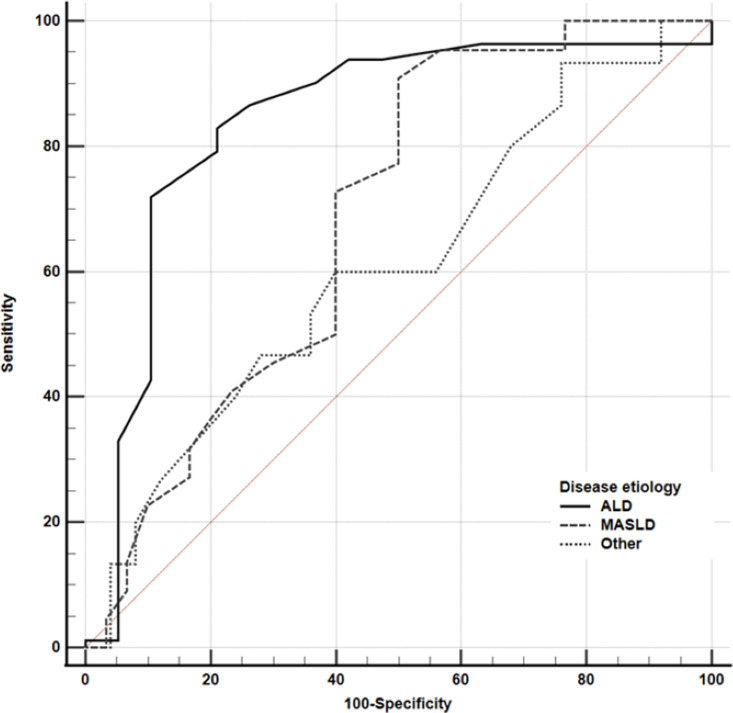
Cross-sectional analysis of HVPG in the prediction of hepatic decompensation by the disease etiology. For ALD (solid line), MASLD (dashed line), and other etiologies (dotted line), AUROC = 0.826 (0.709-0.943), 0.687 (0.544-0.831), and 0.608 (0.425-0.791). Differences between ALD vs. MASLD (P = 0.147), MASLD vs. other (P = 0.51), ALD vs. other (P = 0.049). Thresholds for hepatic decompensation ALD > 13 mmHg (83,5%, 73.7%), MASLD > 10mmHg (sensitivity 90.9%, specificity 50.0%), and other > 12 mmHg (60.0%, 60.0%).

## Results

### Baseline comparison

In total, we enrolled 220 ACLD patients, with 128 cases with alcohol associated, 52 with metabolic associated and 40 with other etiologies, mainly autoimmune and viral hepatitis. A comparison of the baseline characteristics among the disease etiology groups is displayed in Table 1. Patients with MASLD vs. ALD had similar age (60.4 vs. 56.9 years), a higher proportion of females (67.3% vs. 31.2%), lower Child-Pugh-Turcotte (7.0 vs. 9.0) and MELD scores (13.0 vs. 16.0), and a lower white blood cell count (4.9 vs. 6.15 G/mm^3^). At baseline, the beta-adrenergic blockers were taken in 61.7% of ALD patients, 55.8% of MASLD and 52.2% of other etiologies (P value for trend = 0.5). ALD patients had a lower proportion of compensated patients (14.8%) compared to MASLD (57.7%), and other etiologies (62.5%). (P value < 0.001). The probability of death or liver transplantation within 3 and 6 months did not differ among the groups (Table 1).

### HVPG correlations

Numerical values of HVPG were most correlated with Child-Pugh-Turcotte scores (rho = 0.328, P < 0.001) and serum albumin (rho = -0.323, p < 0.001). A significant correlation was also observed with white blood cells (rho = 0.171, p = 0.012), lymphocyte count (rho = 0.172, p = 0.278), and MELD (rho = 0.134, p = 0.053).

### HVPG by disease phenotype

A comparison of baseline characteristics, HVPG and TIPS placements by the status of compensation is displayed in Table 2. Overall, 74 patients (33.6%) were compensated, variceal bleeding was present in 79 cases (35.9%), refractory ascites in 40 cases (18.2%), and alcoholic hepatitis in 27 cases (12.3%). The median HVPG was 11.0 mmHg among compensated and 16.0, 19.0 and 18.0 mmHg among patients with alcoholic hepatitis, refractory ascites and bleeding, respectively. Death or liver transplantation occurred within 90 and 180 days in 12.2 and 18.9% of compensated cases, 20.3 and 26.6% of cases with bleeding, and 29.6 and 44.4% of patients with alcoholic hepatitis.

### HVPG by disease etiology and phenotype

Values of the HVPG and proportions of gradients above the cut-offs by disease etiology and status of compensation are displayed in Table 3 and Fig 1. Overall, the median HVPG in ALD, MASLD, and other etiologies was 18.0, 14.0, and 11.5 mmHg (P value < 0.01). We observed a decreasing HVPG trend among the etiological groups.

Numerical HVPG values by disease etiology and status of compensation are displayed in Fig 2. In compensated patients, there was no difference in the gradient across the etiologies: 10.0, 11.5, and 11.0 mmHg (P = 0.97) (Fig 2A). In decompensated patients, we observed a decreasing trend from ALD, through to MASLD and other etiologies: 19.0, 15.5, and 14.0 mmHg (P = 0.019) (Fig 2B). The trend was observed regardless of the beta-adrenergic blocker treatment at baseline. Decompensated patients with ALD had significantly higher HVPG than other etiologies, but the numerical difference between ALD and MASLD did not reach statistical significance. Proportions of decompensated patients with HVPG values above the relevant cut-offs are compared in Fig 3.

In the subgroup of 51, 16 and 12 decompensated patients with variceal bleeding the median HVPG was 19.0, 15.5, and 13.5 mmHg with a decreasing trend among the groups (P = 0.014). The differences between HVPG values did not reach statistical significance. The TIPS was placed in 34 (66.6%), 9 (56.2%) and 3 (25%) of patients (P = 0.25).

In the subgroup of 31, 6 and 3 decompensated cases with refractory ascites, the median HVPG was 19.0, 16.5, and 16.0 mmHg (P = 0.57). The differences between HVPG values did not reach statistical significance. The TIPS was placed in 27 (87.1%), 5 (83.3%), and 1 (33.3%) patient (P = 0.09).

### HVPG cut-offs

In a cross-sectional analysis, we explored the HVPG threshold for the presence of hepatic decompensation by comparing the area under the ROC curve (AUROC). For ALD, MASLD, and other etiologies the AUROC was 0.826 (0.709-0.943), 0.687 (0.544-0.831), and 0.608 (0.425-0.791), respectively (Fig 4). Differences among the etiological groups were not statistically significant with the exception of the difference between ALD and other disease etiologies (P = 0.049). The threshold for hepatic decompensation for ALD, MASLD, and other etiologies was > 13 mmHg (sensitivity 83,5%, specificity 73.7%), > 10mmHg (90.9%, 50.0%), and > 12 mmHg (60.0%, 60.0%).

## Discussion

### The main findings of our study

In our cirrhosis registry cohort of 220 patients, we compared three etiological groups for HVPG values: alcohol (ALD), metabolic associated (MASLD), and other (autoimmune and viral). The groups slightly differed in the baseline characteristics with MASLD and other groups having a higher proportion of females, lower prognostic scores, lower inflammatory markers, and a significantly lower prevalence of disease decompensation compared to the ALD group. In compensated patients, we observed no differences in HVPG among the three groups. In contrast, we observed a significant and gradual decrease in HVPG in the given order of etiologies (i.e., ALD – MASLD – Other). Decompensated ALD patients displayed a higher prevalence of HVPG values above 16 mmHg, particularly those having variceal bleeding. However, the observed numerical differences in HVPG reached statistical significance only between alcohol and other etiologies, but not between alcohol and MASLD. An equally high (>95%) proportion of decompensated ALD or MASLD patients had clinically significant portal hypertension.

### Interpretation

First, our study shows the decreasing trend of HVPG among ALD, MASLD, and inflammatory-derived cirrhosis. Since we observed no differences in compensated disease, the observed trend is likely driven by processes behind decompensation. Due to the relatively low proportions of decompensated patients in the MASLD and Other etiology groups, the limited statistical power could not bring the observed trend into the statistically significant numerical differences in HVPG values. Although the median difference between ALD and MASLD of 3.5 mmHg (mean difference 1.7 mmHg) would likely be statistically significant with higher statistical power, it would point to the theoretically higher numerical HVPG in ALD patients. This could be explained by the dynamic component of portal hypertension, but our data do not support the hypothesis that inflammation in alcoholic hepatitis drives the increased HVPG (Table 2). However, the small difference in HVPG among the etiological groups would not be clinically relevant. The clinically validated threshold of 10 mmHg has been observed in almost all decompensated patients with ALD and MASLD.

Second, our study could highlight the technical and mechanistic/pathophysiological issues behind differences in the degree of HVPG-prognosis link; as HVPG is an indirect marker of portal pressure, and geography of the maximum injury to the acinus differ between ALD and MASLD, HVPG readings could underestimate true portal pressure in MASLD (see below).

Third, the proportion of patients taking beta-adrenergic blockers was surprisingly low. The retrospective nature of the study conducted before the current recommendation was in place, and the inclusion criteria of hospitalization for HVPG measurement could explain this observation. Nevertheless, there is a need for thorough auditing and promotion of the use of these medications in the future.

Fourth, despite our results add to the notion of different HVPG in different etiologies of ACLD and lend support to the continued research into the pathophysiology of portal hypertension, from the clinical point of view they confirm the etiology-independent cut-off 10 mm Hg for CSPH as defined by BAVENO Consensus.

In the literature, evidence for etiology-specific liver hemodynamic parameters has been reported. In a study dated in the 1980s, authors compared values of the true portal pressure with the wedged hepatic pressure and demonstrated that the difference was higher in idiopathic or “non-alcoholic cirrhosis” exceeding 4 mmHg on average [13]. Later studies have also consistently reported that patients with steatohepatitis compared with those with chronic hepatitis C had lower HVPG for each stage of fibrosis. They suggested that marked perisinusoidal fibrosis contributed to the real portal pressure by adding a pre-sinusoidal component that is not captured by the wedged hepatic vein pressure [14]. In decompensated patients with viral hepatitis, the HVPG accurately reflects the true portal pressure [15]. However, in MASLD cirrhosis, a recent study has confirmed that the discordance of more than 10% was common – which was more than in other etiologies (37.5 vs. 14%, p = 0.003) [16]. Another study indicated that hepatic steatosis is not a major factor contributing to portal venous pressure in patients with chronic liver disease. They suggested that other factors contributing to portal hypertension - such as increases in intrahepatic resistance due to hepatic inflammation/fibrosis, as well as splanchnic vasodilatation and the hyperdynamic circulation in patients with CSPH might have had a much stronger impact on portal pressure than hepatic steatosis itself [17]. In our previous study, we have found higher HVPG and more inflammation in ALD yet the prognosis was worse in MASLD patients; at that time, we sought the potential explanation in two domains: 1) in the less reversible stage of fibrosis due to lesser dynamic component of portal hypertension in MASLD; and 2) chronic multiorgan dysfunction as a known downstream consequence of the metabolic syndrome in MASLD patients. On the other hand, abstinence and complex therapy of ALD is well known to immediately and effectively impact dynamic component of portal hypertension which can translate into a better prognosis despite higher baseline HVPG as compared to patients with MASLD.

Finally, a recent multinational cross-sectional study has looked at the threshold of HVPG and the presence of hepatic decompensation in NAFLD vs. hepatitis C cirrhosis. Authors have explored comparable cohorts matched for age, gender, and disease severity. They reported a higher prevalence of hepatic decompensation at any given threshold of HVPG in NAFLD cirrhosis [18]. Evidence from the mentioned studies is firm on the differences between HCV and NAFLD cirrhosis. However, there is not much evidence for the difference in portal pressure and HVPG in alcoholic cirrhosis. A recent study pooled ALD and hepatitis C cirrhosis together but did report an excellent correlation between WHVP and RPP in 40 ALD patients. Even though our study has confirmed a lower HVPG in decompensated MASLD cirrhosis (dACLD), the proportion of CSPH was similarly very high in decompensated disease and variceal bleeding (Fig 3 and Table 3). Although some authors have reported, that hepatic decompensation in MASLD may occur in HVPG lower than 10 mmHg, our findings support the validity of the existing threshold for CSPH of>=10 mmHg as suggested by the BAVENO consensus. Notably, the natural history and pathogenesis of ALD are similar to MASLD, with typical marked perisinusoidal fibrosis in both etiologies [19]; therefore, it is currently unclear whether the presinusoidal component is the sole factor responsible for underestimating the true portal pressure in MASLD patients. It also remains to be elucidated if the lower HVPG readings in otherwise comparably decompensated patients with MASLD represent underestimation or truly lower portal pressure as compared with ALD. Some authors have proposed that obesity could increase the intraabdominal pressure in MASLD patients leading to bouts of increased portal pressure independently of liver fibrosis [20]. Diabetes and hyperlipidemia with increased circulating vasoactive substances and a pro-inflammatory state have also been proposed [21].

Our study has several limitations. First, it is retrospective and biased towards patients undergoing the HVPG measurement which was the inclusion criterion. Second, albeit exploring separately the subgroups provided an interesting insight, we admit limited statistical power for firm conclusions in the subgroup analysis. Third, after we have identified a limited number of patients with dACLD, we pooled the viral and autoimmune groups with similar inflammatory pathogenesis to enable a valid comparison with ALD and MASLD in the entire cohort.

## Conclusion

In conclusion, our study exploring the differences in hepatic venous pressure gradient in advanced chronic liver disease confirmed that *i)HVPG significantly differs according to etiology of ACLD*, particularly in the context of cirrhosis complications. This evidence lends strong support for the more convenient use of HVPG in an etiology-specific/ more personalized manner. *ii)Patients with MASLD-derived dACLD had lower HVPG in comparison to ALD*; this was driven mostly by the subgroup of patients with variceal bleeding. *iii)The threshold for decompensation did not differ between the two groups*; and, an equal proportion of patients with dACLD due to MASLD or ALD had CSPH.

## Abbreviations

HVPG: hepatic venous pressure gradient
MASLD: metabolic dysfunction-associated steatotic liver disease
ALD: alcohol-associated liver disease
ACLD: advanced chronic liver disease
NAFLD: nonalcoholic fatty liver disease
MELD: model for end-stage liver disease
TIPS: transjugular intrahepatic portosystemic shunt
CSPH: clinically significant portal hypertension
HE: hepatic encephalopathy
AKI: acute kidney injury
HCC: hepatocellular carcinoma
LT: liver transplantation
ROC: receiver operating curve
AUROC: area under the receiver operating curve
IVC: inferior vena cava
WHVP: wedged hepatic venous pressure
LFI: liver frailty index
AI: autoimmune liver diseases
RPP: real portal pressure
